# CD40 induces renal cell carcinoma-specific differential regulation of TRAF proteins, ASK1 activation and JNK/p38-mediated, ROS-dependent mitochondrial apoptosis

**DOI:** 10.1038/s41420-019-0229-8

**Published:** 2019-12-04

**Authors:** Khalidah Ibraheem, Albashir M. A. Yhmed, Tahir Qayyum, Nicolas P. Bryan, Nikolaos T. Georgopoulos

**Affiliations:** 1 Department of Biological Sciences, School of Applied Sciences, University of Huddersfield, Huddersfield, UK; 2 Urology Department, Calderdale and Huddersfield NHS Foundation Trust, Huddersfield Royal Infirmary, Huddersfield, UK; 3 Present Address: Department of Medical Laboratory Sciences, University of Sebha, Tripoli, Libya

**Keywords:** Extracellular signalling molecules, Apoptosis, Renal cell carcinoma

## Abstract

A unique feature of CD40 among the TNF receptor (TNFR) superfamily is its exquisitely contextual effects, as originally demonstrated in normal and malignant B-lymphocytes. We studied renal cell carcinoma (RCC) in comparison to normal (human renal proximal tubule) cells, as a model to better understand the role of CD40 in epithelial cells. CD40 ligation by membrane-presented CD40 ligand (mCD40L), but not soluble CD40 agonist, induced extensive apoptosis in RCC cells; by contrast, normal cells were totally refractory to mCD40L. These findings underline the importance of CD40 ‘signal-quality’ on cell fate and explain the lack of pro-apoptotic effects in RCC cells previously, while confirming the tumour specificity of CD40 in epithelial cells. mCD40L differentially regulated TRAF expression, causing sustained TRAF2/TRAF3 induction in RCC cells, yet downregulation of TRAF2 and no TRAF3 induction in normal cells, observations strikingly reminiscent of TRAF modulation in B-lymphocytes. mCD40L triggered reactive oxygen species (ROS) production, critical in apoptosis, and NADPH oxidase (Nox)-subunit p40phox phosphorylation, with Nox blockade abrogating apoptosis thus implying Nox-dependent initial ROS release. mCD40L mediated downregulation of Thioredoxin-1 (Trx-1), ASK1 phosphorylation, and JNK and p38 activation. Although both JNK/p38 were essential in apoptosis, p38 activation was JNK-dependent, which is the first report of such temporally defined JNK-p38 interplay during an apoptotic programme. CD40-killing entrained Bak/Bax induction, controlled by JNK/p38, and caspase-9-dependent mitochondrial apoptosis, accompanied by pro-inflammatory cytokine secretion, the repertoire of which also depended on CD40 signal quality. Previous reports suggested that, despite the ability of soluble CD40 agonist to reduce RCC tumour size in vivo via immunocyte activation, RCC could be targeted more effectively by combining CD40-mediated immune activation with direct tumour CD40 signalling. Since mCD40L represents a potent tumour cell-specific killing signal, our work not only offers insights into CD40’s biology in normal and malignant epithelial cells, but also provides an avenue for a ‘double-hit’ approach for inflammatory, tumour cell-specific CD40-based therapy.

## Introduction

CD40 is a member of the tumour necrosis factor (TNF) receptor (TNFR) superfamily and interaction with its ligand, CD40L (CD154), plays a crucial role in immune responses^1,2^. Yet, apart from hematopoietic cells, such as B cells and antigen-presenting cells, CD40 is also expressed by epithelial cells of various origins, including bladder, liver and ovarian carcinoma cells, normal epithelial cells as well as endothelial cells. There is accumulating evidence that CD40–CD40L signalling may strongly influence non-lymphoid cell fate in addition to inducing cytokine secretion^3^.

CD40 lacks kinase activity and related intracellular signalling motifs, thus utilises adapter molecules TNFR-associated factors (TRAFs) for signal transduction^4^, with TRAF2 and TRAF3 being the main TRAF proteins that play significant and often opposing roles in CD40 signalling^5^. After CD40L-mediated activation, CD40 translocates to lipid rafts, where it associates with various TRAFs^6^ to activate downstream mitogen-activated protein kinases (MAPKs)^7,8^.

Recent studies provided evidence that the consequences of CD40 ligation may differ in normal and malignant cells, thus its effects may be highly context-specific^5^. Moreover, the ‘quality’ of the CD40 signal may determine whether CD40L–CD40 interactions are pro-apoptotic: extensive receptor cross-linking by membrane-presented CD40L (mCD40L) causes extensive apoptosis, while soluble agonists (e.g. agonistic antibodies) cause little apoptosis^9–14^. However, the mechanisms that define these properties of CD40 have only recently started to become investigated^15^.

CD40 expression has been demonstrated in normal renal cells and their malignant counterparts (renal cell carcinoma, RCC). CD40 is expressed by human RCC lines in vitro and its stimulation by soluble agonist triggered secretion of pro-inflammatory cytokines, yet no direct pro-apoptotic effects have been reported^16,17^. Others reported that in primary RCC cultures stimulation of CD40 by soluble agonists caused proliferation and enhanced cell motility^18^. By contrast, in normal proximal tubule cells cultured in vitro CD40 ligation induced both pro-inflammatory cytokine secretion^19^ and anti-inflammatory signals^20^. Therefore, the consequences of CD40 ligation in both normal and RCC cells remain partially controversial and essentially unexplored.

Here, we provide the first detailed investigation on the effect of CD40 ligation in human RCC cells as well as their normal counterparts. We demonstrate that mCD40L induced extensive apoptosis accompanied by pro-inflammatory cytokine secretion in malignant cells, while soluble CD40 agonist is weakly apoptotic. Importantly, normal cells were completely refractory to CD40-mediated killing. CD40 ligation differentially regulates TRAF2 and TRAF3 expression in normal vs. malignant cells and apoptosis induction involves a signalling pathway that entrains ASK1 activation and reactive oxygen species (ROS)-mediated death, with JNK and p38 being sequentially involved in the induction of the intrinsic Bak/Bax-associated mitochondrial apoptotic pathway.

## Results

### CD40 expression by normal and malignant renal cells and its regulation by pro-inflammatory cytokines

**Fig. 1:**
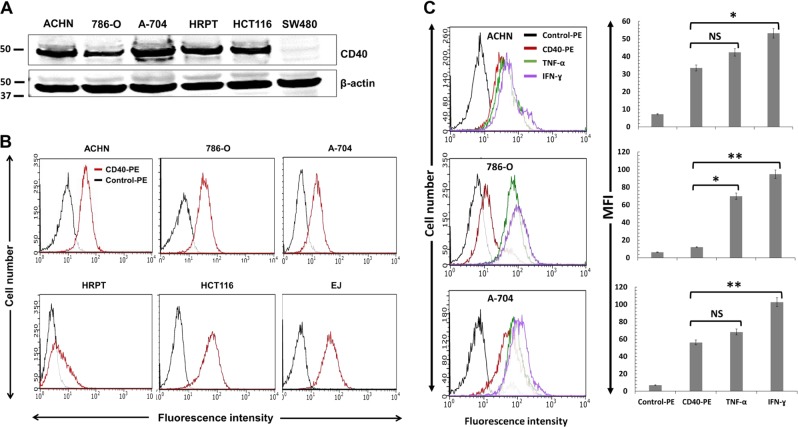
CD40 expression by normal (HRPT) and malignant (RCC) cells and its regulation by pro-inflammatory cytokines. **a** Detection of CD40 expression in RCC lines ACHN, 786-O and A-704 and in normal HRPT cells by immunoblotting. Lysates from HCT116 and SW480 cell cultures served as CD40+ve and CD40-ve controls, respectively. Correct loading (20 µg protein/lane) was confirmed by β-actin detection. **b** Surface expression of CD40 by HRPT cells and RCC lines was examined by flow cytometry using CD40-PE (red histograms) in comparison to control PE-conjugated (black histograms) antibody. HCT116 and EJ cell lines served as CD40+ve controls. **c** RCC cells were treated with TNF-α (green histograms) or IFN-γ (purple histograms) for 48 h and CD40 expression was assessed alongside untreated cells by flow cytometry. Overlay histograms from a representative experiment are shown on the left and a summary of two independent experiments is shown on the right. Bars show mean median fluorescence intensity (MFI) of three technical replicates ± SEM.

To investigate CD40 expression in RCC cells, we used the well-characterised cell lines ACHN, 786-O and A-704 (ref. ^21^). Expression was also examined in primary human renal proximal tubule cells, HRPT. By immunoblotting, all RCC lines were CD40 positive, with the greatest expression observed in A-704 and ACHN, while lower expression was observed in 786-O and in normal (HRPT) cells (Fig. 1a). Expression was compared to HCT116 (CD40 positive) and SW480 (CD40 negative) colorectal carcinoma (CRC) lines^11^.

Flow cytometry corroborated cell-surface CD40 expression (Fig. 1b), in comparison to HCT116 and urothelial cell carcinoma (UCC) line EJ^10^. Pro-inflammatory cytokines can up-regulate CD40 expression in epithelial cells such as urothelial (EJ)^9^ and colorectal (HCT116)^11^. Treatment with TNF-α and particularly IFN-γ increased expression, with the greatest induction observed in 786-O (Fig. 1c). Hence, both normal (HRPT) and malignant (RCC) cells express CD40 and expression can be increased by TNF-α and IFN-γ treatment.

### Membrane-CD40L (mCD40L), but not soluble receptor agonist, induces apoptotic death in maligant (RCC) cells, yet normal renal cells are refractory

**Fig. 2:**
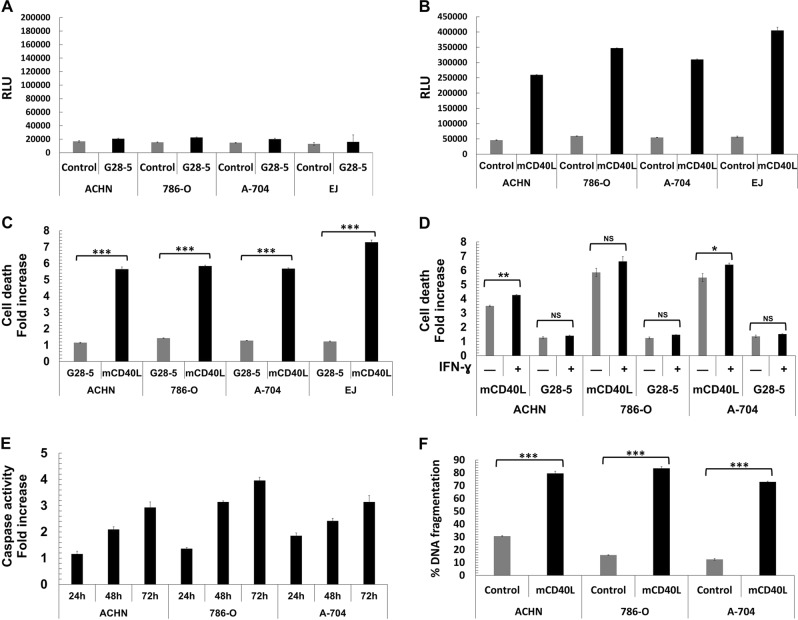
Membrane-CD40L (mCD40L) but not soluble CD40 agonist induces extensive cell death (apoptosis) in RCC lines. **a** ACHN, 786-O, and A-704 cells were either untreated (Control) or treated with 10 µg/ml cross-linked agonistic anti-CD40 mAb (G28-5) for 48 h and death was detected using the CytoTox-Glo assay (see Methods). Results are representative of at least two experiments and are presented as background-corrected relative luminescence unit (RLU) readings. EJ cells were included as positive controls. Bars show mean RLU of 3–4 technical replicates ± SEM. **b** ACHN, 786-O, and A-704 cells were co-cultured with 3T3Neo (Control) or 3T3CD40L (mCD40L) effector cells for 48 h and death was detected using the CytoTox-Glo assay. Results are typical of tive independent experiments and are presented as background-corrected relative luminescence units (RLU). EJ cells served as positive controls for mCD40L-mediated apoptosis. Bars show mean RLU of 5–6 technical replicates ± SEM. **c** Raw data from the experiments shown in (**a**) and (**b**) are presented as Cell death fold increase in RLU relative to control, in order to compare the degree of apoptosis induction for mCD40L vs. G28-5 mAb treatment in all RCC lines (and control EJ cells). Bars show mean fold change ± SEM. **d** RCC cell lines were treated with mCD40L or G28-5 mAb alongside appropriate controls in the presence (+) or absence (–) of IFN-γ (10^3^ units/ml) and cell death was detected using the CytoTox-Glo assay. Results are presented as Cell death fold increase in background-corrected RLU readings relative to control and are representative of two independent experiments. Bars show mean fold change of 5–6 technical replicates ± SEM. **e** RCC lines were treated with mCD40L for 24, 48 and 72 h and effector caspase-3/7 activity was assessed (see Methods). Results are presented as Caspase activity fold increase in background-corrected fluorescence relative to control and are representative of three experiments. Bars show mean fold caspase activity of 5–6 technical replicates ± SEM. **f** RCC lines were treated with mCD40L for 48 h alongside appropriate negative controls (and positive control, staurosporine-treated cells) and DNA fragmentation was assessed (see Methods). Results are presented as % DNA fragmentation relative to positive control (maximal DNA fragmentation) and are representative of at least two independent experiments. Bars show mean % DNA fragmentation of six technical replicates ± SEM.

We initially activated CD40 in the panel of RCC lines using soluble agonist, in particular cross-linked agonistic anti-CD40 G28-5 mAb^11,15^. Treatment caused limited RCC cell death at 48 h, as was observed in positive control EJ (Fig. 2a)—similar results were obtained at earlier/24 h or later/72 h time-points (not shown).

To investigate if signal ‘quality’ (extent of receptor cross-linking) is critical in inducing RCC cell death, we treated the panel of RCC lines with membrane-CD40L (mCD40L). We utilised a culture system^22^ which involves co-culture of target (epithelial) cells with effectors (fibroblasts) expressing mCD40L, alongside non-ligand expressing fibroblasts (Control). We performed pre-titration experiments to (a) determine optimal target:effector ratios (0.6:1, 0.8:1 and 1:1) and (b) assess cell death at different time-points (24, 48, 72 h) post-receptor ligation (Supplementary Fig. 1). mCD40L caused marked cell death by 48 h (0.8:1 ratio) in all RCC lines, comparable to EJ (Fig. 2b), with similar observations at 72 h (Supplementary Fig. 1). Death induction by mCD40L was extensive and several-fold higher than the effect of G28-5 mAb which was minimal (Fig. 2c). In light of its ability to up-regulate CD40 (Fig. 1c), we examined whether IFN-γ could augment soluble agonist (or mCD40L)-mediated effects; IFN-γ could not sensitise RCC cells to G28-5 mAb, although it caused some enhancement in mCD40L-mediated death (Fig. 2d).

**Fig. 3:**
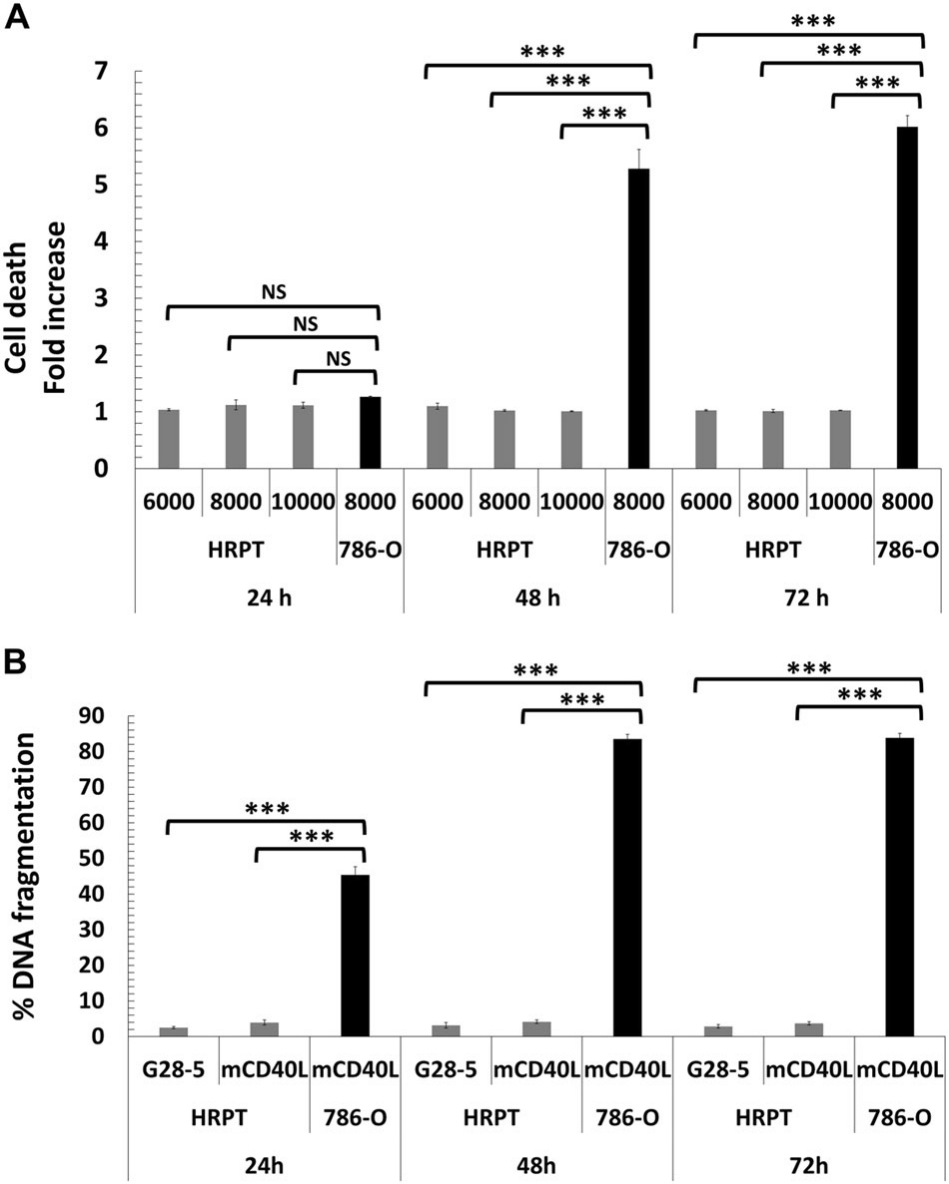
mCD40L is a highly pro-apoptotic signal in malignant (RCC) but not in normal HRPT cells. **a** HRPT cells were co-cultured with 3T3Neo (Control) or 3T3CD40L (mCD40L) effector cells to assess the effect of mCD40L-mediated CD40 ligation. The co-cultures involved a defined number of effector cells (10,000/well) and three different densities of HRPT cells (6000, 8000 and 10,000/well). Cell death was detected at 24, 48 and 72 h using the CytoTox-Glo assay (see Methods). Results are presented as Cell death fold increase in background-corrected RLU readings (mCD40L relative to control) and are representative of three independent experiments. 786-O RCC cells served as positive controls for mCD40L-mediated apoptosis. Bars show mean fold change of 5–6 technical replicates ± SEM. **b** HRPT cells (seeded at 8000 cells/well) were either treated with 10 µg/ml cross-linked agonistic anti-CD40 mAb (G28-5) or mCD40L (by co-culture as above) for 24, 48 and 72 h alongside appropriate negative controls (and positive control, staurosporine-treated cells) and DNA fragmentation was assessed (see Methods). Results are presented as % DNA fragmentation relative to positive control (maximal DNA fragmentation) and are representative of two experiments. 786-O RCC cells served as positive control for mCD40L-mediated DNA fragmentation. Bars show mean % DNA fragmentation of three technical replicates ± SEM.

With regard to the ‘nature’ of mCD40L-killing, in addition to its ability to cause plasma membrane integrity compromisation (Fig. 2a–d), mCD40L caused an increase in effector caspase-3/-7 activity, with activation occuring within 24 h post-ligation (Fig. 2e). In addition, mCD40L triggered extensive DNA fragmentation (Fig. 2f) in RCC lines by 48 h (fragmentation evident at 24 h—also Fig. 3b). These results show that CD40 induces extensive killing in RCC cells, which is dependent on the mode of receptor ligation but irrespective of the level of CD40-positivity, and mCD40L engages a death pathway with classical apoptotic features.

Having demonstrated extensive CD40-induced apoptosis in RCC cells, we tested whether CD40 may cause cytotoxicity to their normal counterparts. When we treated HRPT cells with mCD40L (as above), no apoptosis was detectable, although mCD40L induced apoptosis in 786-O cells (Fig. 3a). Concordantly, neither soluble agonist G28-5 mAb nor (and more importantly) mCD40L induced DNA fragmentation in HRPT cells, in comparison to mCD40L-treated 786-O cells (Fig. 3b). Therefore, although mCD40L induces extensive apoptosis in malignant (RCC) cells, normal cells remain totally refractory to mCD40L.

### CD40 signal ‘quality’ determines repertoire and extent of pro-inflammatory cytokine secretion

**Fig. 4:**
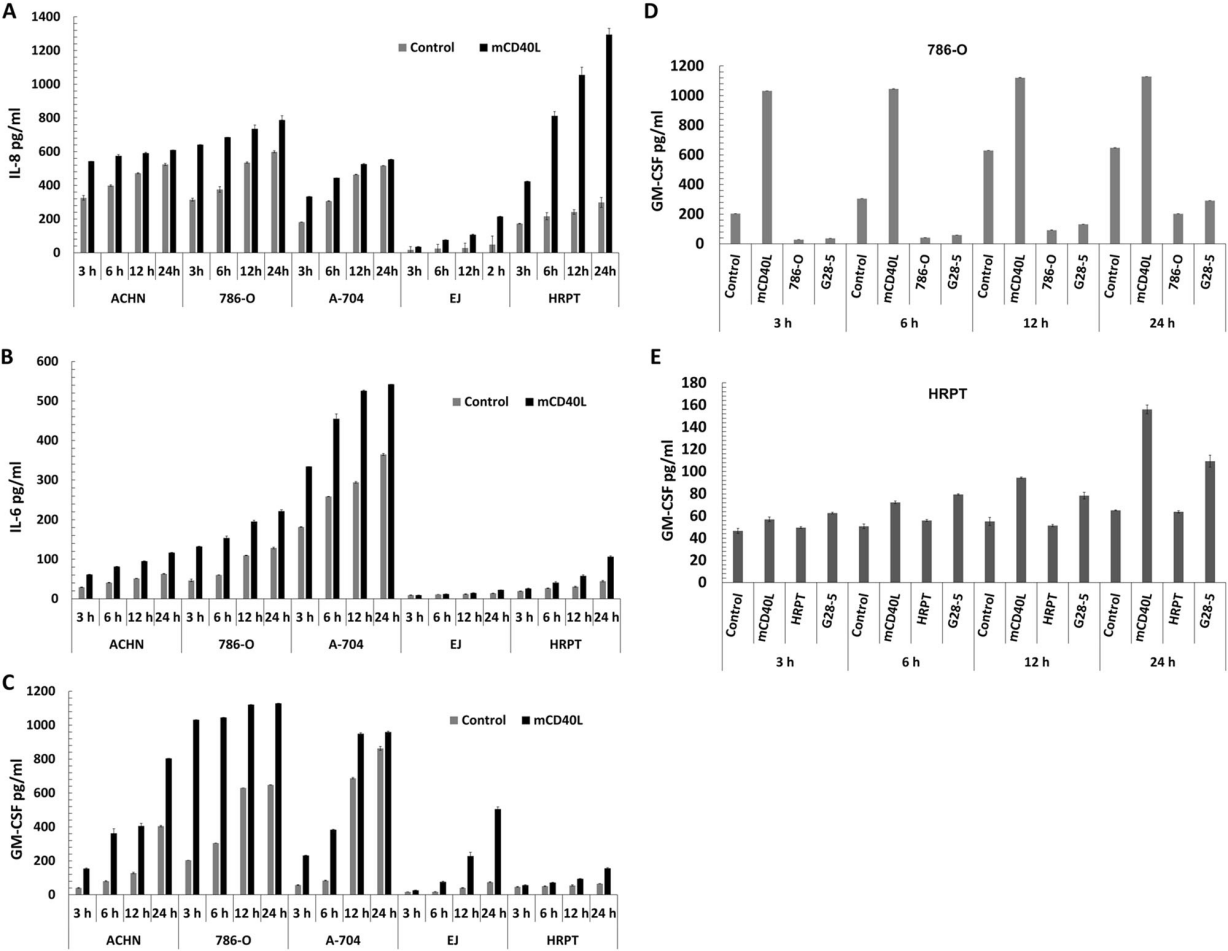
Induction of pro-inflammatory cytokine secretion in human RCC lines vs. normal HRPT cells is dependent on the mode of CD40 ligation. **a**–**c** RCC cell lines ACHN, 786-O and A-704, the positive control EJ and normal HRPT cells were treated with mCD40L. IL-8 (**a**), IL-6 (**b**) and GM-CSF (**c**) secretion in comparison to controls was detected at the indicated time-points post CD40 ligation (3, 6, 12 and 24 h) by ELISA assays (see Methods). Bars represent mean cytokine concentration (pg/ml) ± SEM for three technical replicates and are representative of two independent experiments. **d**, **e** RCC cell line 786-O (**d**) and normal HRPT cells (**e**) were treated with ‘mCD40L’ alongside negative controls (‘Control’), and CD40 was also activated with agonistic anti-CD40 mAb (‘G28-5′), and secretion of GM-CSF was measured by ELISA as above. Untreated RCC cells (‘786-O’ in **d**) and normal cells (‘HRPT’ in **e**), respectively, were also included as background controls. Bars show mean GM-CSF concentration (pg/ml) ± SEM for two technical replicates and are typical of two independent experiments.

Using a membrane-based array approach we determined global cytokine/chemokine secretion induced by mCD40L in 786-O RCC cells compared to control EJ (Supplementary Fig. 2). The cytokines/chemokines mostly induced in both 786-O and EJ cells were interleukin-8 (IL-8), IL-8-related chemokine GRO-α, IL-6, MCP-1/CCL2 and granulocyte-macrophage colony-stimulating factor (GM-CSF). We then quantified secretion of IL-8, IL-6 and GM-CSF in RCC and in normal (HRPT) cells by enzyme-linked immunosorbent assay (ELISA). Despite basal levels of IL-8, mCD40L rapidly augmented IL-8 secretion in all RCC lines (3–6 h) (Fig. 4a). IL-8 induction was striking in HRPT cells, where mCD40L continued to induce secretion after 12 h (Fig. 4a). mCD40L caused gradual induction of IL-6 in all RCC lines, although this was less-pronounced in normal cells (Fig. 4b). Interestingly, although both RCC and normal cells secreted low basal levels of the cytokine, mCD40L rapidly and markedly induced GM-CSF secretion in RCC cells and, albeit to a lesser extent, in HRPT cells (Fig. 4c).

Although IL-8 secretion is induced by soluble CD40 agonist, GM-CSF secretion may only be triggered by mCD40L^11^. We therefore compared GM-CSF secretion in malignant (786-O) and normal (HRPT) cells following CD40 ligation by G28-5 mAb and mCD40L. mCD40L caused rapid GM-CSF induction in 786-O cells that peaked at 3 h post-ligation, whereas soluble agonist caused little induction of GM-CSF even after 12 h (Fig. 4d). Concordantly, when we compared soluble vs. mCD40L in normal HRPT cells, we found that mCD40L caused more marked GM-CSF secretion than did soluble agonist (Fig. 4e). Therefore, mCD40L induces rapid and sustained pro-inflammatory cytokine secretion, compared to soluble agonist, both in RCC and normal cells, despite its differential effects in cell fate.

### Differential regulation of TRAF2 and TRAF3 by mCD40L in normal vs. malignant cells

**Fig. 5:**
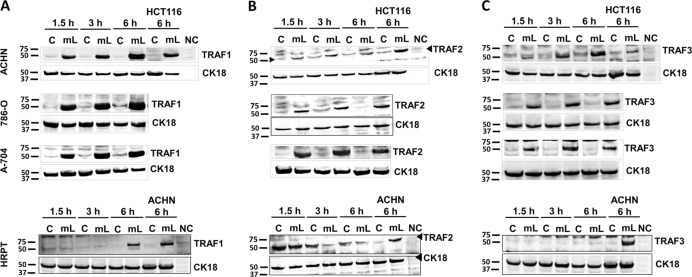
Differential regulation of TRAF proteins by mCD40L in normal (HRPT) and malignant (RCC) cells. RCC lines ACHN, 786-O and A-704, and normal HRPT cells were treated with mCD40L for the indicated time periods (1.5, 3 and 6 h) and expression of TRAF1 (**a**), TRAF2 (**b**) and TRAF3 (**c**) was detected by immunoblotting (40 µg protein/lane) in controls (‘C’) vs. mCD40L-treated cells (‘mL’). Equal loading for human epithelial cell lysate was confirmed by CK18 detection (see Methods). As positive controls for TRAF1 (**a**), TRAF2 (**b**) and TRAF3 (**c**) protein expression induction, in RCC cell immunoblotting experiments (top panels) lysates from HCT116 cells that were treated with control (‘C’) or treated with mCD40L (‘mL’) for 6 h were included, while for HRPT cell experiments (bottom panels) lysates from ACHN cells untreated or treated with mCD40L for 6 h were used. Lysate from effector (3T3CD40L) cells alone (20 µg protein/lane) served as negative control (NC) and confirmed the human-protein specificity of the anti-TRAF1 (**a**), anti-TRAF2 (**b**) and anti-TRAF3 (**c**) antibodies.

To investigate mCD40L-triggered signalling in renal cells, we initially focused on receptor-proximal events and assessed TRAF protein regulation. mCD40L caused rapid upregulation of TRAF1 in all RCC lines, in comparison to low basal expression. Induction of TRAF1 was also evident in HRPT cells, although this response was slower (observed after 6 h) and less-pronounced when compared to RCC cells (Fig. 5a).

We then investigated expression of the most ‘central’ TRAFs in CD40 signalling, TRAF2 and TRAF3. We observed rapid and marked TRAF2 induction in all RCC lines; yet, interestingly, in HRPT cells, which expressed basal TRAF2 protein levels, mCD40L caused TRAF2 downregulation (Fig. 5b). Equally strikingly-differential was the effect of mCD40L on TRAF3 expression, as in malignant cells mCD40L caused rapid, marked and sustained TRAF3 upregulation, whereas no induction of TRAF3 expression was detectable in normal (HRPT) cells (Fig. 5c). Therefore, mCD40L regulates early signalling events in renal cells and, more importantly, TRAF2 and TRAF3 regulation is fundamentally different between normal and malignant cells.

### mCD40L induces apoptosis along a signalling axis involving direct JNK-mediated p38 phosphorylation

**Fig. 6:**
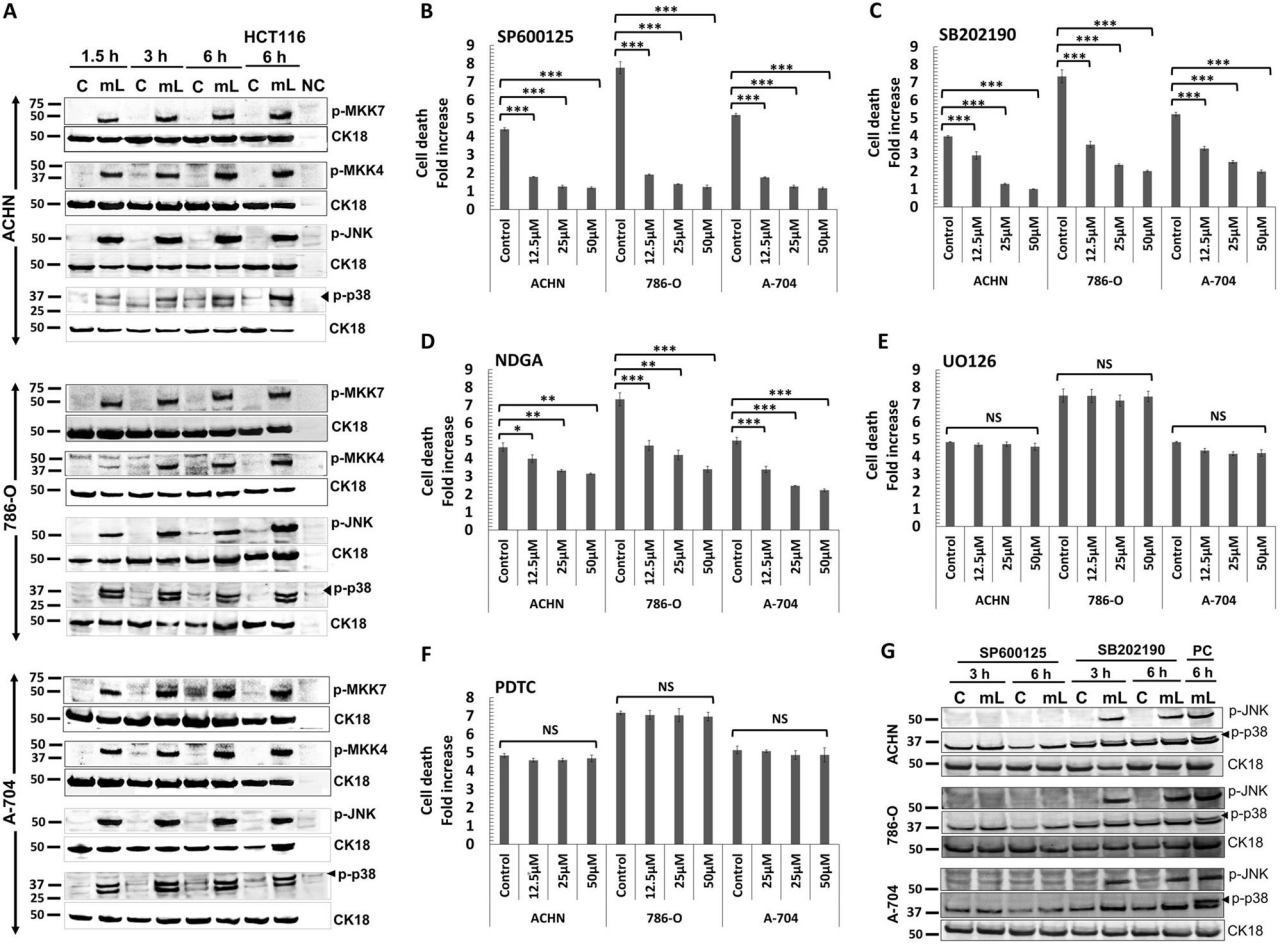
Regulation of intracellular signalling pathways and their functional role in mCD40L-mediated tumour cell apoptosis. **a** ACHN, 786-O and A-704 cells were treated with mCD40L for the indicated time periods (1.5, 3 and 6 h) and expression of phosphorylated-MAPKs MKK4 (p-MKK4), MKK7 (p-MKK7), JNK (p-JNK) and p38 (p-p38) was detected in controls (‘C’) vs. mCD40L-treated cells (‘mL’) by immunoblotting (40 µg protein/lane). Equal loading for human epithelial cell lysate was confirmed by CK18 detection (see Methods). As positive controls for p-MKK4/7, p-JNK and p-p38 protein expression induction, lysates from HCT116 cells that were treated with control (‘C’) or treated with mCD40L (‘mL’) for 6 h were included. Lysate from effector (3T3CD40L) cell monocultures served as negative control (NC) and confirmed the human-protein specificity of the antibodies. **b**–**f** ACHN, 786-O and A-704 cells were treated with mCD40L in the absence (vehicle control—denoted ‘Control’) or presence of the indicated concentration (12.5, 25 and 50 μM) of JNK inhibitor SP600125 (**b**), p38 inhibitor SB202190 (**c**), AP-1 inhibitor NDGA (**d**), MEK/ERK inhibitor U0126 (**e**) and NF-κB inhibitor (PDTC). Cell death was detected 48 h later using the CytoTox-Glo assay (see Methods). Results are presented as Cell death fold increase in background-corrected RLU readings relative to control (mCD40L treatment vs. controls) and are representative of three independent experiments. Bars show mean fold change of 5–6 technical replicates ± SEM. **g** ACHN, 786-O and A-704 cells were treated with mCD40L for the indicated time periods (3 and 6 h) in the presence of 25 μM JNK inhibitor SP600125 or p38 inhibitor SB202190 and expression of phosphorylated-MAPKs JNK (p-JNK) and p38 (p-p38) was detected in controls (‘C’) vs. mCD40L-treated cells (‘mL’) by immunoblotting (40 µg protein/lane). ACHN, 786-O and A-704 cells treated with mCD40L for 6 h in the absence of inhibitor (vehicle controls) were also included (denoted as positive control, ‘PC') for each experiment. Equal loading for human epithelial cell lysate was confirmed by CK18 detection (see Methods).

Pharmacological blockade of MEK/ERK and NF-κB had no effect on mCD40L-mediated death. By contrast, AP-1 inhibition attenuated cell death, while JNK blockade fully abrogated mCD40L-mediated killing (Fig. 6). As these findings functionally implicated the JNK/AP-1 pathway in apoptosis, we examined the expression of key components of this signalling axis. mCD40L induced phosphorylation of both MKK4 and MKK7, as well as triggering sustained phosphorylation of downstream target JNK (Fig. 6a).

p38 has a well-documented role in stress-related responses in the kidney and has been implicated in renal cell apoptosis triggered by TNF-α^23^, yet no involvement in CD40-mediated apoptosis has been reported^10^. p38 inhibitor SB202190 attenuated mCD40L-apoptosis (Fig. 6c), as effectively as did the JNK inhibitor SP600125 (Fig. 6b). Concordantly, mCD40L triggered p38 phosphorylation and this response was sustained (Fig. 6a).

Interestingly, the induction of p-p38 lagged behind the activation of p-JNK, as JNK phosphorylation plateaued by 1.5 h whereas maximal p-p38 expression was evident at later time-points (3 h in A-704 or 6 h in ACHN cells) (Fig. 6a). To discover whether JNK and p38 operated independently (but the timing of activation differed) or whether JNK caused downstream p38 activation, we treated RCC cells with mCD40L in the presence of JNK or p38 inhibitor and p-JNK and p-p38 expression was assessed. p38 inhibitor SB202190 attenuated p-p38 induction (Fig. 6g) but had no effect on the induction of p-JNK expression. However, the JNK inhibitor SB600125 completely-suppressed both JNK and p38 phosphorylation (Fig. 6g). Therefore, mCD40L-mediated apoptosis involves both the JNK/AP-1 and p38 pathways, but p38 activation appears to be JNK-mediated.

### mCD40L induces the mitochondrial apoptotic pathway

**Fig. 7:**
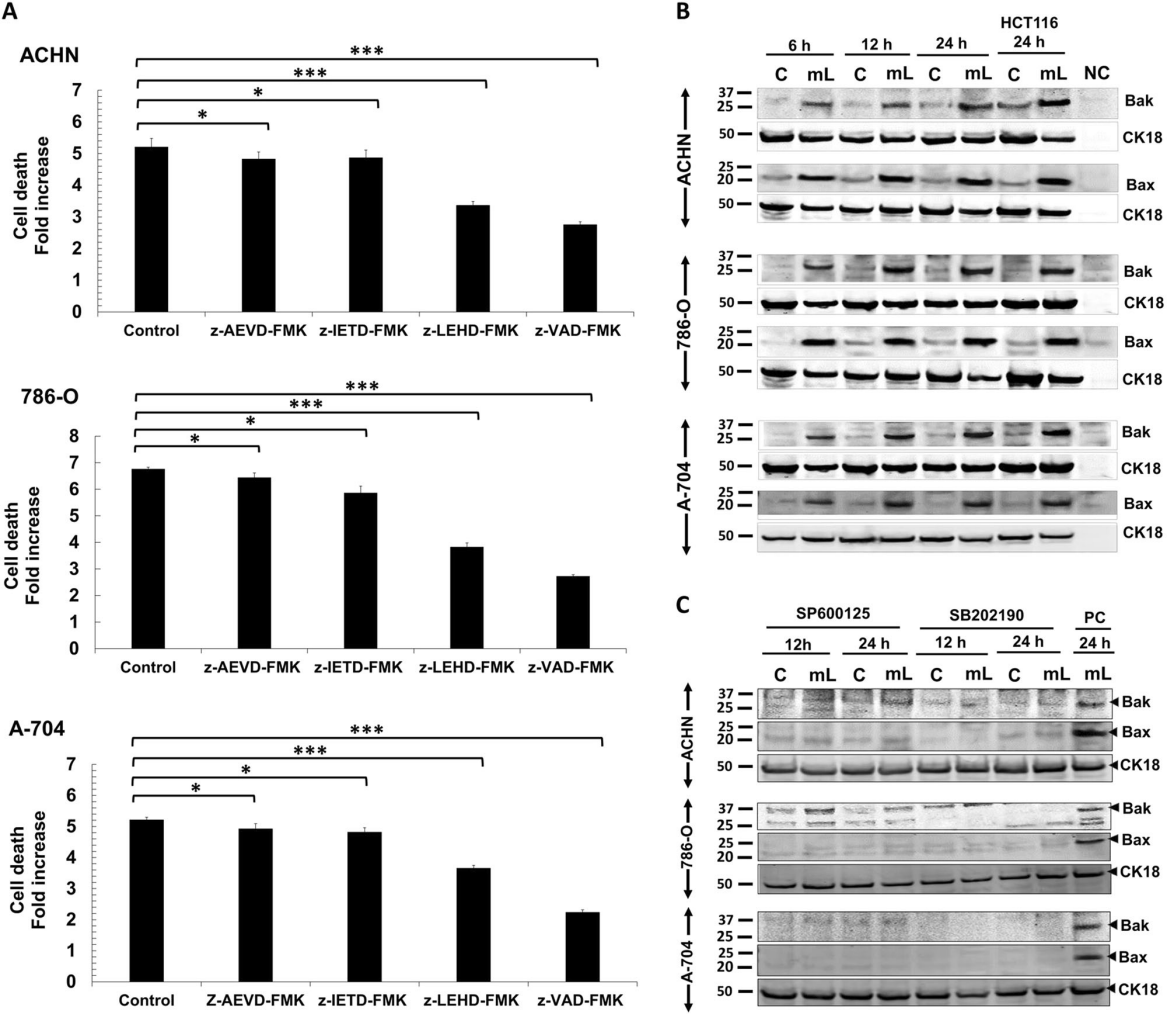
Role of caspase activation and induction of the mitochondrial (intrinsic) pathway during mCD40L-mediated tumour cell apoptosis. **a** ACHN, 786-O and A-704 cells were treated with mCD40L in the absence (vehicle control—denoted ‘Control’) or presence of 100 μM of inhibitor of caspase-10 (z-AEVD-FMK), caspase-8 (z-IETD-FMK), caspase-9 (z-LEHD-FMK) or pan-caspase inhibitor (z-VAD-FMK). Cell death was detected 48 h later using the CytoTox-Glo assay (see Methods). Results are presented as Cell death fold increase in background-corrected RLU readings relative to control (mCD40L treatment vs. controls) and are representative of three independent experiments. Bars show mean fold change of 4–6 technical replicates ± SEM. **b** ACHN, 786-O and A-704 cells were treated with mCD40L for the indicated time periods (6, 12 and 24 h) and expression of Bak and Bax was detected in controls (‘C’) vs. mCD40L-treated cells (‘mL’) by immunoblotting (40 µg protein/lane). Equal loading for human epithelial cell lysate was confirmed by CK18 detection (see Methods). As positive controls for Bak and Bax protein expression induction, lysates from HCT116 cells that were treated with control (‘C’) or treated with mCD40L (‘mL’) for 24 h were included. Lysate from effector (3T3CD40L) cells alone served as negative control (NC) and confirmed the human-protein specificity of the antibodies. **c** ACHN, 786-O and A-704 cells were treated with mCD40L for the indicated time periods (12 and 24 h) in the presence of 25 μM JNK inhibitor SP600125 or p38 inhibitor SB202190 and expression of Bak and Bax was detected in controls (‘C’) vs. mCD40L-treated cells (‘mL’) by immunoblotting (40 µg protein/lane). ACHN, 786-O and A-704 cells treated with mCD40L for 24 h in the absence of inhibitor (vehicle controls) were also included (denoted as positive control, ‘PC') for each experiment. Equal loading for human epithelial cell lysate was confirmed by CK18 detection (see Methods).

Having demonstrated mCD40L-mediated effector caspase (-3/7) activation (Fig. 2e), biochemical inhibition of initiator caspase-8 and -10 showed little effect on mCD40L-killing, even though the caspase-8 biochemical inhibitor effectively blocked TRAIL-mediated cell death (not shown). By contrast, caspase-9 blockade markedly reduced death and pan-caspase inhibitor nearly completely blocked apoptosis (Fig. 7a). This suggested that mCD40L-mediated apoptosis is caspase-dependent and apoptosis may entrain the ‘intrinsic’/mitochondrial pathway. We used whole-cell lysates and assessed whether mCD40L induced Bak and Bax, the key regulators of mitochondrial outer membrane permeabilisation (MOMP), cytochrome *c* release and caspase-9 activation^24^. We could detect basal Bak and Bax expression in all RCC lines but mCD40L triggered marked induction of Bak and particularly Bax expression 6 h post-ligation (Fig. 7b) (no induction observed <3 h—not shown). Bax levels plateaued more rapidly, whereas Bak induction was gradual until expression peaked 24 h post-treatment. Interestingly, blockade of the JNK/AP-1 and p38 pathways fully abrogated induction of both Bax and Bak (Fig. 7c). Therefore, mCD40L-mediated death in RCC cells is caspase-dependent and involves JNK/p38-mediated induction of the mitochondrial apoptotic pathway.

### mCD40L activates ASK1 and the NADPH oxidase (Nox) complex and induces ROS-dependent apoptosis

**Fig. 8:**
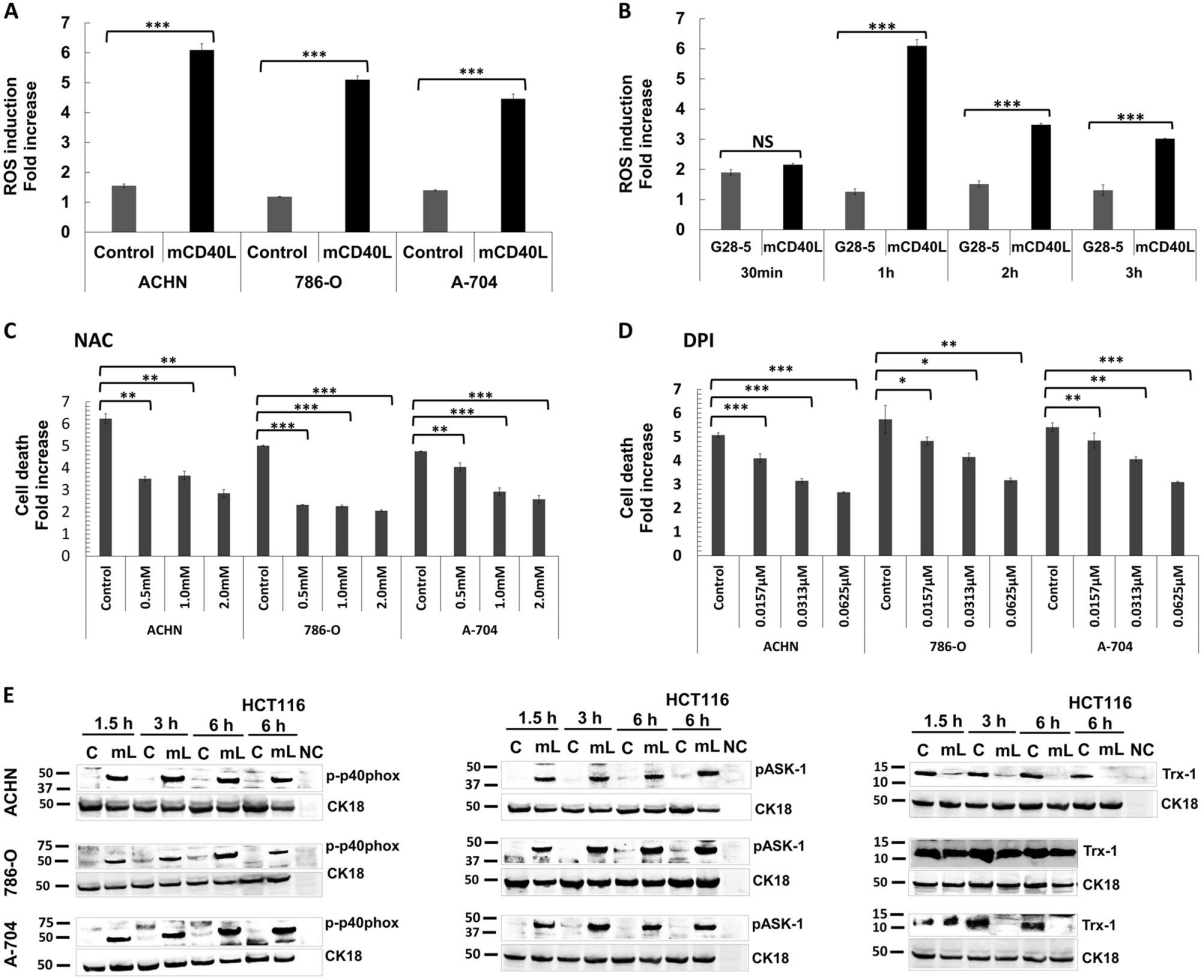
ROS generation by mCD40L, but not soluble CD40 agonist, and activation of ROS-dependent signalling for apoptosis induction. **a** ACHN, 786-O and A-704 cells were co-cultured with 3T3Neo or 3T3CD40L effector cells for 1 h and intracellular ROS was detected by treatment with 1 μM H2DCFDA (see Methods). Background-corrected relative fluorescence unit (RFU) readings obtained were used to present the data as ROS induction fold increase, which is fold change in RFU detected for H2DCFDA-treated 3T3Neo/RCC cell (‘Control’) and 3T3CD40L/RCC cell (‘mCD40L’) co-cultures vs. untreated co-cultures. Results are representative of three independent experiments. Bars show mean fold change of 5–6 technical replicates ± SEM. **b** To compare the differences in the extent of ROS generation for mCD40L vs. soluble agonist, ACHN cells were treated with ‘mCD40L’ (alongside negative controls) or agonistic anti-CD40 mAb (‘G28-5′) for the indicated time periods (30 min, 1 h, 2 h and 3 h) and ROS levels were detected as in **a**. For mCD40L treatments, data are presented as background-corrected RFU readings of mCD40L relative to controls; for G28-5 treatments, data are presented as RFU readings relative to untreated cells. Results (ROS induction fold increase) are representative of two independent experiments. Bars show mean fold change of 4–5 technical replicates ± SEM. **c**, **d** ACHN, 786-O and A-704 cells were treated with mCD40L in the absence (vehicle control—denoted ‘Control’) or presence of the indicated concentration of inhibitors NAC (**c**) and DPI (**d**). Cell death was detected 48 h later using the CytoTox-Glo assay (see Methods). Results are presented as Cell death fold increase in background-corrected RLU readings relative to control (mCD40L treatment vs. controls) and are representative of three independent experiments. Bars show mean fold change of 4–5 technical replicates ± SEM. **e** ACHN, 786-O and A-704 cells were treated with mCD40L for the indicated time periods (1.5, 3 and 6 h) and expression of phospho-p40phox, phospho-ASK1 and Trx-1 was detected in controls (‘C’) vs. mCD40L-treated cells (‘mL’) by immunoblotting (40 µg protein/lane). Equal loading for human epithelial cell lysate was confirmed by CK18 detection (see Methods). As positive controls, lysates from HCT116 cells that were treated with control (‘C’) or treated with mCD40L (‘mL’) for 6 h were included. Lysate from effector (3T3CD40L) cell monocultures served as negative control (NC) and confirmed the human-protein specificity of the antibodies.

As activation of JNK by TNFRs can be ROS-dependent^25^, we detected ROS production in RCC cells. mCD40L caused rapid ROS release (30 min) and levels peaked at 1 h (Fig. 8a); thereafter, ROS levels remained high (Supplementary Fig. 3). By contrast, non-apoptotic G28-5 mAb induced modest changes in ROS (Fig. 8b). Induction of ROS was critical in apoptosis, as the ROS scavenger *N*-acetyl l-cysteine (NAC) markedly attenuated mCD40L-mediated death (Fig. 8c).

Apoptosis signal-regulating kinase 1 (ASK1) MAP3K acts upstream of p38/JNK and its activation is ROS-mediated^26^. mCD40L triggered phosphorylation of ASK1 in all RCC lines (Fig. 8e, right panels), the timing of which was in line with ROS induction (Fig. 8b). Notably, ROS induction occurred far sooner than apoptosis induction (Fig. 2); apart from the mitochondria, the other source of ROS for cell signalling functions is the NADPH oxidase (Nox) complex^27^. mCD40L triggered phosphorylation of the regulatory Nox-2 subunit p40phox (Fig. 8e) and Nox inhibition using diphenyleneiodonium (DPI) suppressed mCD40L-mediated death (Fig. 8d).

Because ASK1 activation occurs following ‘release’ from its inhibitor Thioredoxin (Trx), facilitated by ROS-mediated Trx oxidation^26^, we explored the effect of mCD40L on Trx-1. Notably, in untreated RCC cells we observed high basal Trx-1 expression, with expression being most pronounced in the most rapidly proliferating 786-O cells (Fig. 8e). Intriguingly, mCD40L rapidly attenuated Trx-1 protein expression either fully (ACHN and A-704 cells) or partially (786-O) (Fig. 8e). Therefore, mCD40L-mediated RCC death is ROS-dependent, pro-apoptotic signalling entrains activation of Nox as well as ASK1 activation, accompanied by Trx-1 downregulation.

## Discussion

A unique feature of CD40 among TNFR members^28^ is its exquisitely contextual influence on cell fate; the consequences of CD40 ligation appear to be different in normal and malignant B-lymphocytes, yet recent evidence suggests that this may also apply to epithelial cells. In parallel, the ‘quality’ of the signal may determine whether CD40L–CD40 interactions are pro-apoptotic; mCD40L causes apoptosis, while soluble agonists are mainly growth-inhibitory^9–13,29^. The molecular signalling pathways underpinning these two fundamental properties of CD40, i.e. the TRAF members involved, MAPK pathways invoked and death mechanisms employed, have only recently become the subject of detailed investigation^15^.

Our study provides the first systematic investigation of the effects of CD40 ligation in RCC cells as well as compared these effects to normal (HRPT) cells. Normal and malignant cells expressed CD40 which could be up-regulated by TNF-α/IFN-γ. We found that soluble CD40 agonist had little effect; however, mCD40L induced extensive killing, irrespective of the level of CD40-positivity. Cross-linked soluble agonist (G28-5 mAb) even in combination with IFN-γ could not trigger death in RCC cells. mCD40L-mediated death had apoptotic features, including plasma membrane compromisation, DNA fragmentation and caspase-3/-7 activation, and caspase activity was essential for apoptosis. Collectively, these findings highlighted the importance of the mode of CD40 ligation in determining functional outcome in carcinoma cells^10,11^, and provided an explanation for lack of detectable pro-apoptotic effects in RCC cells in previous studies which employed soluble CD40 agonists^16–18^.

Due to its strong pro-apoptotic effect in malignant cells, it was essential to investigate the effect of mCD40L in normal (HRPT) cells. Previous studies reported that soluble CD40 agonists stimulated IL-8 and MCP-1 induction and MAPK signalling^19^, but did not cause cell death in HRPT cells in vitro, despite ROS induction^20^. We have now shown that despite being highly pro-apoptotic in RCC cells, mCD40L remains non-apoptotic in HRPT cells, thus normal cells are refractory to CD40-killing. This provides for the first time evidence for a tumour cell-specific pro-apoptotic effect in RCC, as well as adding support to previous observations that mCD40L is non-apoptotic in normal human uro-epithelial cells^15^.

CD40 signalling has been well-characterised in the context of B-cell function, where the role of the TRAFs in activating signalling pathways, including NF-кB, JNK/AP-1 and p38, has been elegantly studied by Bishop et al.^30,31^. CD40-mediated TRAF modulation and subsequent MAPK signalling in normal and malignant epithelial cells is far less characterised^10,15^. Pro-apoptotic CD40 ligation differentially regulated TRAF expression in renal cells; mCD40L induced TRAF1 expression; however, in normal cells TRAF1 induction was slower. Most dramatically different was the regulation of TRAF2/3; mCD40L caused their rapid and sustained induction in RCC cells, yet in HRPT cells we observed downregulation of TRAF2 and no TRAF3 induction. Our observations in normal cells are strikingly reminiscent of the regulation of TRAFs in normal B-lymphocytes, where TRAFs 2 and 3 are either downregulated or not activated^32–34^ and corroborate for the first time previous observations in normal uro-epithelial cells^10^. As there is evidence that in UCC and CRC cells TRAF3 plays a critical role in mCD40L-mediated apoptosis by triggering JNK activation and apoptosis^10,12^, it is tempting to speculate that TRAF3 might play an equally important role in RCC cell apoptosis. However, previous studies reported no clear patterns of TRAF2 regulation^10^; intriguingly, in RCC cells mCD40L induced TRAF2 as effectively as TRAF3, and future studies should address the possible functional role/interplay of TRAFs 2/3 in CD40 signalling in RCC cells.

MAPKKs such as MKK4/MKK7 can trigger death-inducing JNK activation^35,36^ and JNK/AP-1 induction underpins mCD40L-mediated death in UCC cells^10,15^; however, p38 activation has never been directly implicated in CD40-mediated carcinoma cell death. We now show that mCD40L caused phosphorylation of MKK4/MKK7 followed by JNK and p38 phosphorylation, and activation of both JNK and p38 was essential in CD40-mediated apoptosis in RCC cells. Although it has been widely reported that JNK/p38 can be simultaneously activated but act independently for apoptosis induction^37^, during mCD40L-mediated apoptosis in RCC cells, p38 activation was fully dependent on JNK activity, suggesting JNK as the direct p38 activator. To our knowledge, this is the first report for such a temporally defined interplay between these two key MAPKs during any known apoptotic programme.

Bcl-2 family proteins Bak/Bax form oligomers to induce MOMP and subsequently apoptosis^38^. Bak and particularly Bax were induced in RCC lines, induction was sustained during apoptosis and was JNK/p38-controlled. ROS are products of mitochondrial metabolism, but also influence cell proliferation/survival^39^. Inability to control ROS by antioxidant pathways causes oxidative stress leading to cellular damage^40^, therefore ROS generation represents ‘proliferation-at-a-cost’ for tumour cells^41^, rendering them susceptible to a ‘lethal pro-apoptotic threshold’^42^. Unlike soluble agonist, mCD40L triggered rapid ROS production in RCC lines which was sustained and was critical in cell death. Interestingly, ROS production occurred far sooner than apoptosis induction; another major ROS source is the Nox complex^27^ and studies in B cells have implicated the regulatory Nox-2 subunit p40phox in CD40 signalling^43^. The demonstration that p40phox is activated by mCD40L and blockade of Nox abrogated apoptosis implies that the initial ‘wave’ of ROS may be the Nox-dependent. These findings suggest a role of Nox in CD40 signalling in RCC cells and support the recent observation of Nox-/ROS-dependent mCD40L-mediated apoptosis in UCC cells^15^.

ROS can trigger apoptosis via activation of JNK^25^ upstream of which is the ‘redox-sensor’ ASK1, normally inhibited by Trx-1. ROS elevation causes Trx-1 release, permitting ASK1 oligomerization and full-activation^26^ and MKK4/MKK7 phosphorylation to subsequently activate JNK and trigger apoptosis. mCD40L caused ASK1 phosphorylation in RCC cells linked with ROS induction, and in line with the MKK4/MKK7 phosphorylation observed, indicating ASK1 as the upstream inducer of JNK, which in turn mediates p38 activation. Thus, mCD40L triggers an ASK1–MKK4/7–JNK–p38 signalling pathway concomitantly with activation of the ROS-generating Nox complex and cell death is ROS-dependent. Interestingly, high basal intracellular Trx-1 expression was detected in RCC lines, supportive of the reported over-expression of the Trx antioxidant pathway in carcinomas^44^. Notably, we detected substantial amounts of soluble Trx-1 protein in RCC cell culture supernatants (data not shown), supporting Trx-1 over-expression. Strikingly, mCD40L rapidly suppressed Trx-1 expression in RCC cells, which could facilitate ASK1 activation. As Trx-1 expression is under the control of the master-regulator Nrf2 (ref. ^45^), future studies should determine if mCD40L-mediated signalling can modulate Nrf2 to control Trx-1 levels.

Inflammatory responses triggered by cancer-cell death linked with induction of ‘immunogenic cell death' to prime/enhance T-cell responses against cancer-cell-derived tumour-associated antigens are regarded essential for cancer therapy^46,47^. In CRC and UCC cells, soluble agonist and mCD40L caused IL-6 and IL-8 secretion; however, only mCD40L induced GM-CSF release^11^. Soluble CD40 agonists can mediate cytokine secretion in RCC cells, particularly MCP-1 (refs. ^16,17^), but mCD40L triggered more marked secretion of several cytokines, while GM-CSF secretion was specifically triggered by mCD40L in normal and RCC cells. Thus, in addition to defining the pro-apoptotic capacity of CD40 ligation, signal ‘quality’ also determines the repertoire/extent of pro-inflammatory cytokine secretion in renal cells.

GM-CSF is a pleiotropic cytokine that enhances recruitment of neutrophils and macrophages towards tumours and assists in tumour cell lysis via macrophages and dendritic cells (DC)^48^; therefore, GM-CSF release concomitantly with mCD40L-mediated apoptosis may be of therapeutic value. A number of important studies by Wiltrout and colleagues provided evidence for the therapeutic potential of CD40 in RCC. They demonstrated that CD40 expression in RCC is linked with prolonged patient survival^49^. Moreover, CD40 activation resulted in recruitment of monocytes and T cells into established RCC tumours in vivo where agonistic mAb increased the presence of DC and caused reduction in tumour size^17^; these effects were immune-mediated, assisted by tumour-cell cytokine secretion and independent of tumour CD40-status. Importantly, however, it was suggested that RCC tumours could be targeted more effectively by combining CD40-mediated immune activation together with delivery of the CD40 signal to the tumour itself. In light of the ability of mCD40L, but not soluble CD40 agonist, to provide a potent tumour cell-specific killing signal, our work has not only offered insights into the underpinning biology of CD40’s effects in normal and malignant epithelial cells, but has also provided a novel avenue for an improved, ‘double-hit’ approach for inflammatory, tumour cell-specific CD40-based approach for cancer therapy.

## Materials and methods

### Cell culture

ACHN, 786-O and A-704 lines were from the ATCC, supplied via Sigma (Sigma, Dorset, UK) or LGC Standards (LGC, Middlesex, UK) and were adapted for culture in DR medium (1:1 v/v mixture of DMEM and RPMI), supplemented with 5% fetal bovine serum (FBS) (Sigma). EJ and HCT116 lines were cultured as previously^22^. 3T3Neo and 3T3CD40L fibroblasts were maintained in DR/10% FBS and DR/10% FBS supplemented with 0.5 mg/ml G418 (Invivogen, supplied by Source BioScience LifeSciences), respectively, with omission of the antibiotic during co-culture experiments (below). Human renal proximal tubule epithelial cells (HRPTEpiC) (Caltag Medsystems, Bucks, UK), referred to as HRPT, were maintained in EpiCM supplemented with FBS, epithelial cell growth supplement and penicillin/streptomycin as recommended by the supplier.

### CD40 ligation

For activation of CD40 by soluble agonist, epithelial cells were treated with G28-5 mAb cross-linked with goat anti-mouse IgG antibody (Sigma)^10,15^. Unless otherwise stated, cells were seeded at 0.8 × 10^4^ cells/well in 96-well plates or 5 × 10^4^ cells/well in 24-well plates, and following overnight incubation they were treated with G28-5 mAb at 10 µg/ml cross-linked with 2.5 µg/ml goat anti-mouse Ig for 48 h. For CD40 activation by mCD40L, 3T3neo (control) and mCD40L-expressing 3T3CD40L (mCD40L) fibroblasts (effector cells) were growth-arrested by treatment with 10 µg/ml of Mitomycin C (MMC; Santa Cruz, supplied by Insight Biotechnology, Middlesex, UK) for 2 h in DR/5% medium and seeded either in 96-well plates at 1 × 10^4^ cells/well for apoptosis detection assays, 10 cm^2^ culture dishes at 3 × 10^6^ cells/dish for protein lysate preparation, or 24-well plates at 6 × 10^4^ cells/well for culture-supernatant collection. Following overnight incubation, culture medium was removed and epithelial cells were added at 0.8 × 10^4^ cells/well in 96-well plates, 3 × 10^6^ in 10 cm^2^ dishes, or 5 × 10^4^ cells/well in 24-well plates, respectively^22^.

### Detection of cell death

In accordance with published guidelines regarding use and interpretation of assays for monitoring cell death^50,51^, a minimum of two assays were routinely utilised for detection of cell death (apoptosis). This involved use of (a) CytoTox-Glo assay (Promega, Southampton, UK) for detection of compromisation of cell membrane integrity, (b) SensoLyte Homogenous AFC Capase-3/7 assay (Anaspec, Cambridge Bioscience, Cambs, UK) for effector caspase-3/7 activation, and (c) DNA Fragmentation ELISA (Roche Diagnostics, West Sussex, UK) for detection of fragmented DNA in culture supernatants. Full experimental details on the use of these assays for the co-culture system of effector/3T3 and target/epithelial cells have been described recently elsewhere^22^.

### Immunoblotting (western blot)

Whole-cell lysates were prepared from epithelial cells cultured alone or from co-cultures with 3T3Neo (controls) and 3T3CD40L (mCD40L-treated) cells, and were denoted as ‘C’ and ‘mL’, respectively, in the figures. Lysate from effector (3T3CD40L) cells alone served as a negative control (denoted as NC). Lysates were separated by 4–12% SDS-PAGE, and electroblotting performed using Immobilon-FL PVDF membrane (Thermo Fisher Scientific, Loughborough, UK) as detailed elsewhere^22^. For epithelial cell-alone lysates, antibodies used were CD40 (sc-13128) (Insight Biotechnology) and β-actin (A5441) (Sigma). For co-culture lysates, antibodies used were TRAF1 (sc-7186), TRAF2 (sc-876) and TRAF3 (sc-949) (Insight Biotechnology); phospho-MKK4 (#4514), phospho-MKK7 (#4171), phospho-ASK1 (#3765), phospho-JNK (#9255), phospho-p38 (#9255), Thioredoxin-1 (#2285S) and phospho-p40phox (#4311) (Cell Signalling Technologies, supplied by New England Biolabs, Herts, UK); Bak (AF816) and Bax (2282-MC-100) (R&D Systems). Antibody binding was detected using goat anti-rabbit IRDye® 800 (Tebu-bio, Cambs, UK) or goat anti-mouse Ig Alexa Fluor 680 (Thermo Fisher Scientific). For lysates prepared from 3T3neo/3T3CD40L (effector) and epithelial (target) cell co-cultures, sample loading was corrected/adjusted for epithelial cells according to reactivity with human-specific anti-cytokeratin 18 (CK18) antibody (C8541) (Sigma) and not using antibodies detecting non-phosphorylated intracellular signalling mediators, as detailed elsewhere^15^. Immunolabelling was visualised using an Odyssey Infra-Red imaging system (LiCor, Cambs, UK).

### Functional inhibition studies using pharmacological inhibitors

Inhibitors for AP-1 (NDGA), NF-κB (PDTC), NADPH oxidase (Nox) (DPI) and the antioxidant *N*-acetyl l-cysteine (NAC) were from Sigma. Inhibitors for JNK (SP600125), p38 (SB202190) and MEK/ERK (U0126) were from Enzo. Inhibitors for caspase-8 (z-IETD-FMK), -9 (z-LEHD-FMK), -10 (z-AEVD-FMK) and pan-caspase inhibitor (z-VAD-FMK) were from R&D Systems (supplied by Bio-Techne, Abingdon, UK). NAC was reconstituted in DR/5% medium and its pH adjusted to 7.0 using 1 M NaOH solution, and was filter-sterilised before use. All other inhibitors were reconstituted in DMSO (Sigma). RCC cells were co-cultured with 3T3Neo or 3T3CD40L in the presence of inhibitors for 48 h and apoptosis determined as above. Cells treated with DMSO alone (vehicle controls) were included.

### Cytokine secretion detection

Epithelial cells were either (a) cultured in 24-well plates at 5 × 10^4^ cells/well and treated with 10 µg/ml of cross-linked G28-5 mAb or (b) co-cultured at 5 × 10^4^ cells/well with 6 × 10^4^ cells/well growth-arrested 3T3CD40L and 3T3Neo in 24-well plates. Culture supernatants were collected at specified time-points post-receptor ligation, and secretion of IL-6, IL-8 and GM-CSF was measured by cytokine-specific ELISA or membrane array-based detection (R&D Systems/Bio-Techne). For ELISA assays measurements were made spectrophotometrically and for membrane arrays by fluorescence measurements on an Odyssey Infra-Red imaging system (LiCor), as recommended by the manufacturer.

### Flow cytometry

For detection of CD40 expression, cells were cultured in flasks until approximately 80% confluent. Alternatively, for treatment with cytokines, cells were seeded in 24-well plates at 5 × 10^4^ cells/well and, after overnight incubation, they were treated with fresh medium containing 10^3^ units/ml TNF-α or IFN-γ (R&D Systems/Bio-Techne) for 48 h. Cells were then harvested, washed and re-suspended in FACS buffer^22^. CD40 expression was detected using PE-conjugated mouse anti-human CD40 antibody and an isotype control IgG1-PE (BD Biosciences, Berks, UK). In all, 10,000 events were acquired on a Guava EasyCyte flow cytometer and results analysed using EasyCyte software (Millipore, Watford, UK).

### Detection of ROS

For detection of intracellular ROS levels, cells were treated with 1 μM H2DCFDA (Thermo Fisher Scientific) for 30 min, fluorescence measurements (relative fluorescence units, RFU) were taken spectrophotometrically and results expressed as fold increase in RFU, as described in detail previously^22^. It is noted that when co-cultures were performed, effector (3T3Neo/3T3CD40L) cells were not growth-arrested using MMC to minimise background 3T3 cell-associated fluorescence.

### Statistics

Mean values and standard error of the mean (SEM) were used as descriptive statistics. Two-tailed, paired or non-paired Student’s *t*-tests were used for evaluation of statistical significance. For graphical purposes in the figure captions: *p < 0.05, **p < 0.01 and ***p < 0.001, while NS denotes non-significance (*p* > 0.05).

## Supplementary information

Supplementary Information Text

Supplemental Figure 1

Supplemental Figure 2

Supplemental Figure 3
