# Supplementary material for: CD40 induces renal cell carcinoma-specific differential regulation of TRAF proteins, ASK1 activation and JNK/p38-mediated, ROS-dependent mitochondrial apoptosis
Source: Cell Death Discov. 2019 Dec 4;5:148. doi: 10.1038/s41420-019-0229-8 (PMC6892818; doi:10.1038/s41420-019-0229-8)
Supplement: Supplementary file 1 — Supplementary Information Text [file 41420_2019_229_MOESM1_ESM.docx]

# SUPPLEMENTARY INFORMATION

# FIGURE CAPTIONS

## Supplementary Figure 1

## Determination of optimal cell density and time-point for detection of mCD40L-mediated apoptosis in RCC lines

A) RCC cells ACHN, 786-O and A-704 were co-cultured in 96-well plates at 3 different densities (6x10^3^, 8x10^3^ and 1x10^4^ cells/well) with 1x10^4^ cells/well growth-arrested (MMC-treated) effector 3T3Neo (‘Control’) and 3T3CD40L (‘mCD40L’) cells. Cell death was detected at 24, 48 and 72h using the CytoTox-Glo assay (see Methods in main text). Results are typical of at least 2 independent experiments and presented as background-corrected relative luminescence units (RLU). Bars show mean RLU of 5-6 technical replicates ± SEM.

B) Raw data for ACHN, 786-O and A-704 cells from the experiments shown in (A) are presented as Cell death Fold increase in background-corrected RLU readings for mCD40L relative to Control for each cell density and time-point. Bars represent mean fold change ± SEM.

(For statistical analysis see Methods in main text).

## Supplementary Figure 2

## Detection of mCD40L-mediated cytokine secretion in 786-O RCC cells using a human cytokine array

A) 786-O cells were co-cultured with 3T3neo (N+786-O) or 3T3CD40L (L+786-O) effector cells in 24-well plates and culture supernatants were collected at 48h post CD40 ligation. Cytokine secretion was determined using the Human Cytokine Array Panel A (see Methods in main text), which allows detection of a panel of 36 cytokines and chemokines. As a positive control, culture supernatants were also collected from EJ co-cultures with 3T3neo (N+EJ) or 3T3CD40L (L+EJ) effector cells. Cytokine release in supernatants was measured by analyte binding to nitrocellulose membranes and binding was detected according the manufacturer’s instructions. Cytokine detection was visualised by scanning of each membrane using an Odyssey Infra-Red imaging system (using the appropriate secondary antibody recommended by the manufacturer).

B) Detection of fluorescence for each analyte detected (in A) was quantified by densitometry analysis and the results for the most highly-induced cytokines and chemokines (IL-8, IL-6, GM-CSF, GRO-α, sICAM-1 and MCP-1/CCL2) in mCD40L-treated (L+) versus Control (N+) 786-O and EJ cells are presented. Bars show mean fluorescence intensity of the 2 replicate spots for the selected analyte on each membrane array ± SEM.

C) The raw data shown in (B) are presented as fold increase in fluoresce intensity in mCD40L relative to control (‘Cytokine Secretion Fold increase’) in order to allow better comparison for mCD40L-mediated cytokine/chemokine induction between 786-O and EJ cells. Bars represent mean fold increase in secretion for the selected analyte ± SEM.

## Supplementary Figure 3

## Detection of mCD40L-mediated ROS generation in RCC lines

ACHN, 786-O and A-704 cells were co-cultured with 3T3Neo or 3T3CD40L effector cells for the indicated time periods (30min, 1h, 2h and 3h) and intracellular ROS levels were detected by treatment with 1μM H2DCFDA (see Methods in main text). Background-corrected relative fluorescence unit (RFU) readings obtained were used to present the data as ROS induction Fold increase, which is fold change in RFU detected for H2DCFDA-treated 3T3Neo/RCC cell (‘Control’) and 3T3CD40L/RCC cell (‘mCD40L’) co-cultures versus untreated co-cultures. Results are typical of 3 independent experiments. Bars show mean fold change of 4-6 technical replicates ± SEM.

(For statistical analysis see Methods in main text).
