# Supplementary figures and images for: CD40 induces renal cell carcinoma-specific differential regulation of TRAF proteins, ASK1 activation and JNK/p38-mediated, ROS-dependent mitochondrial apoptosis
Source: Cell Death Discov. 2019 Dec 4;5:148. doi: 10.1038/s41420-019-0229-8 (PMC6892818; doi:10.1038/s41420-019-0229-8)

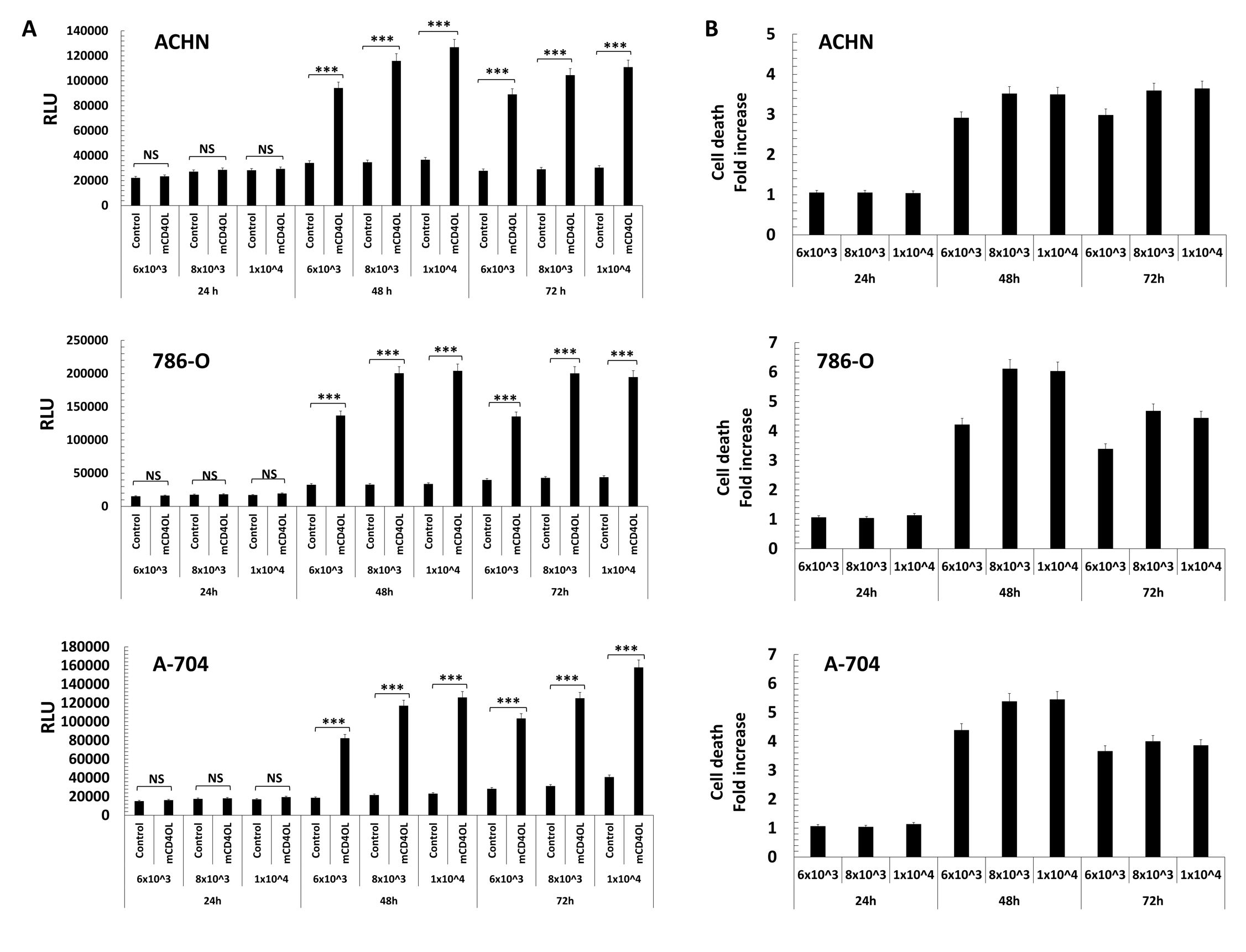

Supplement: Supplementary file 2 — Supplemental Figure 1 [file 41420_2019_229_MOESM2_ESM.jpg]

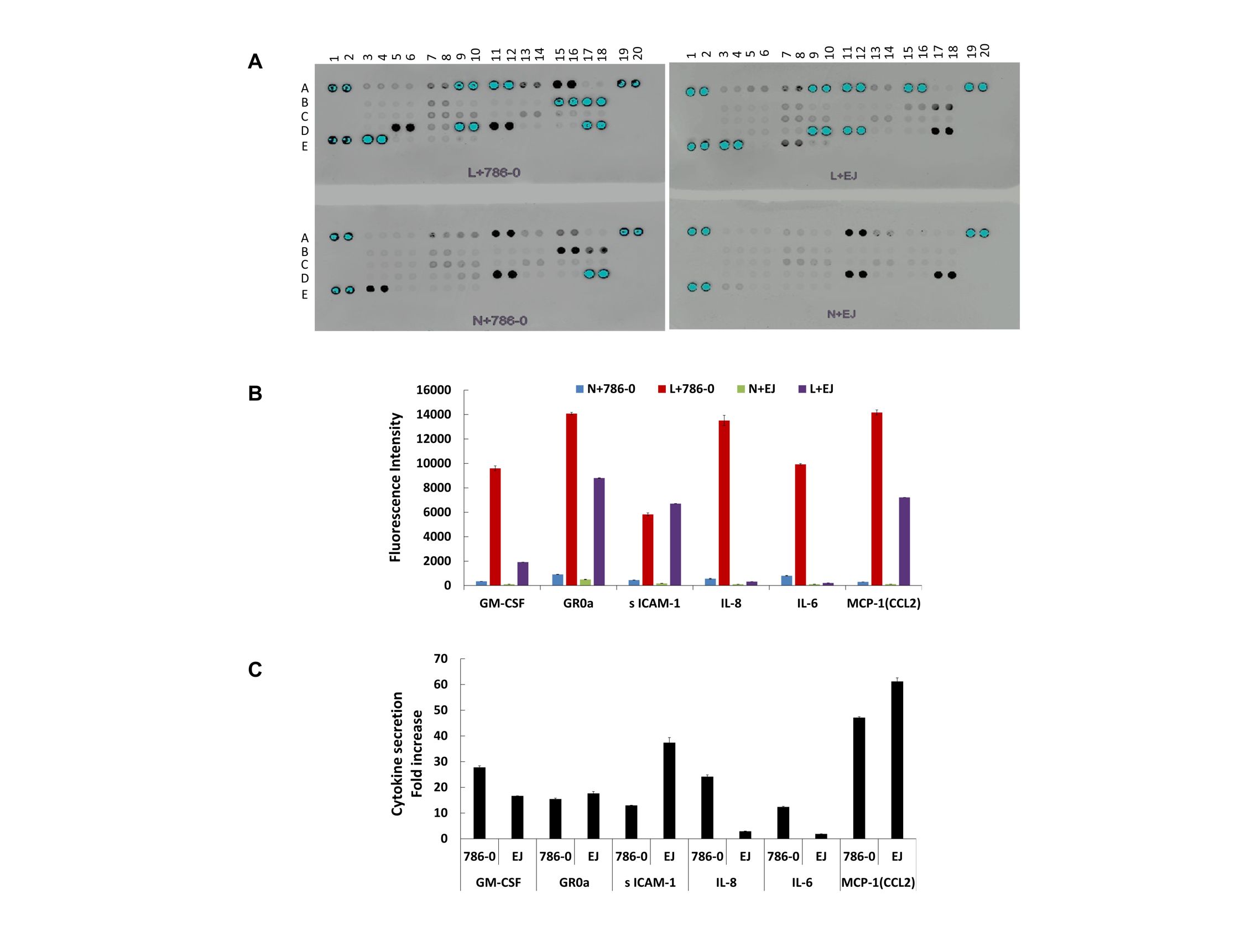

Supplement: Supplementary file 3 — Supplemental Figure 2 [file 41420_2019_229_MOESM3_ESM.jpg]

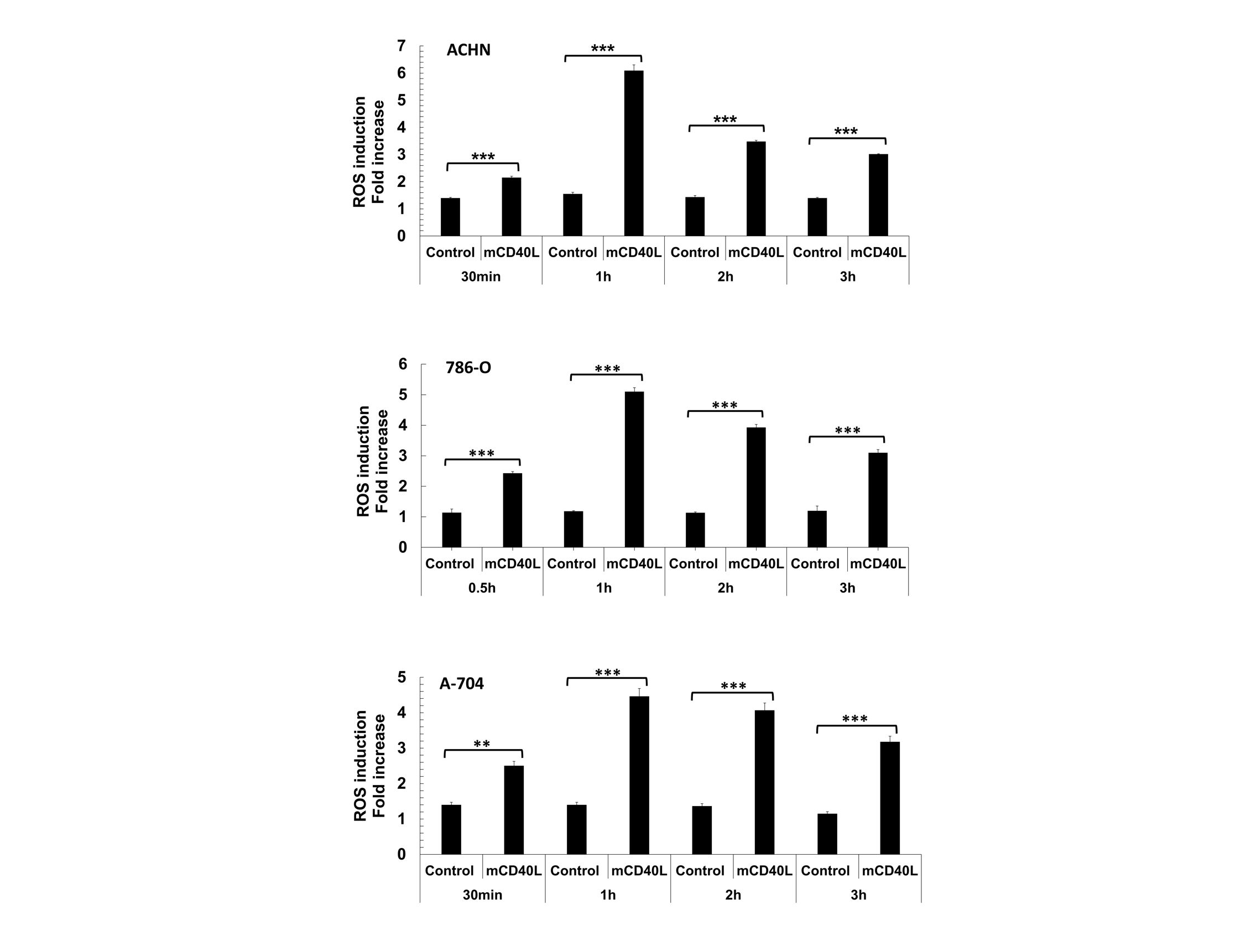

Supplement: Supplementary file 4 — Supplemental Figure 3 [file 41420_2019_229_MOESM4_ESM.jpg]
